# Variation in the reported prevalence of Huntington’s disease: a systematic review and guide to interpretation

**DOI:** 10.1007/s00415-025-13255-1

**Published:** 2025-07-24

**Authors:** Alexander Thompson, Oliver Quarrell, Mark Strong

**Affiliations:** 1 Present Address: School of Medicine and Population Health, University of Sheffield, Sheffield, UK; 2 Sheffield Children’s Hospital, Sheffield, UK

**Keywords:** Huntington’s disease, Prevalence, Heterogeneity, Meta-analysis, Prediction interval

## Abstract

There is significant variation in the reported estimates of Huntington’s disease (HD) prevalence in different settings. This systematic review was undertaken to describe and assess the sources of heterogeneity in estimated prevalence values, and to consider the role of quantitative synthesis in the context of such heterogeneity. Observational studies from which a prevalence estimate (point or period) or cumulative incidence of HD could be calculated between 1993 and 2024 were sought from Medline and Embase databases. The study features are described and the sources of heterogeneity are discussed. A meta-regression was conducted including predictor variables: continent, median age of population, number of years since 1993, case ascertainment method, and Healthcare Access and Quality Index score. 43 studies met the inclusion criteria. Significant clinical and methodological heterogeneity between studies is described, including differences in case definitions and ascertainment methods, and in the estimates of disease burden calculated. There were differences in the estimated point prevalence between regions and populations within regions, while the estimated point prevalence was shown to be increasing since 1993. Wide prediction intervals in the overall pooled point prevalence (95% prediction interval: 0.32–37.55 cases per 100,000), and the European pooled point prevalence (95% prediction interval: 1.64–19.18 cases per 100,000), indicate the scale of heterogeneity between studies and settings. While such heterogeneity currently limits the validity and utility of quantitative synthesis, developing an accepted consensus on the minimum standards and reporting requirements for HD prevalence studies could reduce the methodological heterogeneity between future studies, enabling more valid and meaningful quantitative synthesis in future.

## Introduction

Huntington’s disease (HD) is a well-recognized autosomal dominantly inherited progressive neurodegenerative disorder; characterized by a movement disorder (often chorea), together with disturbances of cognition and affect, resulting from an expansion of a trinucleotide repeated sequence within the first exon of the *HTT* gene [1–3].

In recent years, much interest has been given to describing the burden of HD in different populations [4–8]. HD can have a significant health and social care impact on both affected individuals and families, and the wider health service [9, 10]. The accurate description and prediction of changes in the burden of the disease in a population is, therefore, of public health significance. In epidemiological terms, the burden of a disease can be described using different measures. In the HD literature, these have typically included point prevalence, period prevalence, and cumulative incidence. These provide related but distinct measures of disease burden, with their definitions summarized in Table 1. In this manuscript, we use the term ‘prevalence’ broadly to describe disease burden in a population. Where specific estimates are referenced, we specify the measure of disease burden used where possible.

**Table 1:** Different measures of disease burden

Measure of disease burden	Mathematical formula	Description
Point Prevalence	$\frac{\text{Number of existing cases at a specific point in time}}{\text{Total population at that specific point in time}}$	Proportion of the population with the disease at a specific moment in time
Period Prevalence	$\frac{\text{Number of cases }\left(\text{existing and new}\right)\text{ during a specific period in time }}{\text{Total population during a specific period in time}}$	Proportion of the population with the disease at any time during a specified period
Cumulative Incidence	$\frac{\text{Number of new cases during a specific period in time }}{\text{Population at risk at the start of the specific period in time}}$	Proportion of at-risk individuals who develop the disease over a specified period

Meta-analysis refers to the statistical combination of results from multiple studies to produce a pooled estimate [11]. While historically meta-analyses have been used to pool results of multiple randomized controlled trials to produce an overall estimate of treatment effect, in recent years, the methodology has been increasingly applied to studies measuring the prevalence of disease [12]. The summary value reported is a pooled prevalence estimate—a weighted average—of all prevalence estimates synthesized. Indeed, in a recent systematic review and meta-analysis the ‘global prevalence’ of HD was reported as 4.88 per 100,000 (95% confidence interval (CI): 3.38–7.06) [7]. However, the apparently high heterogeneity in the prevalence of HD (described by large I^2^ values) between different populations, and at different times, raises questions about the validity and meaningfulness of such a quantitative synthesis [13]. Such levels of heterogeneity in the context of studies measuring treatment effects would ordinarily preclude statistical synthesis [11].

The I^2^ statistic is a proportional value used to describe the level of heterogeneity between studies in a meta-analysis. It addresses the question: ‘*what percentage of the observed variation in prevalence is due to real differences (i.e., heterogeneity) between studies, rather than sampling error?*’ High values of I^2^ are ubiquitous in meta-analyses of prevalence [12]. The observational studies of disease prevalence typically have large sample sizes (often the whole population), resulting in precise estimates of prevalence with low sampling error. The proportional nature of the I^2^ statistic means that as the sampling error tends towards zero, the I^2^ statistic tends towards 100%. This is irrespective of whether heterogeneity between studies is large or small in absolute terms. Consequently, caution has been advised in excluding pooled estimates on the basis of large I^2^ values alone [14]. Prediction intervals have been suggested as a more conservative method of incorporating uncertainty into a meta-analysis where true heterogeneity is expected [15], although only a prediction interval of HD in Latin America has been formally presented to date [8].

Clinical heterogeneity in the estimated prevalence of HD in different populations results from multiple factors. The underlying genetics of the population under investigation has been shown to be correlated with disease prevalence, including: mean CAG repeat length, presence of different genetic haplotypes, and CCG polymorphisms [16–18]. Further, while HD can occur at almost any age, the majority of patients develop HD between 35 and 55 years. Therefore, the age-distribution of the population has implications for the prevalence of disease. Since the estimated prevalence of HD is purportedly increasing in parts of the world, the date of any prevalence studies may impact on prevalence values reported [19].

Methodological heterogeneity may also impact the estimates of disease prevalence. Countries with less developed healthcare services, or countries with higher stigma around disease diagnosis, may diagnose fewer cases [20]. Additionally, study designs benefitting from an active case finding component, in which secondary cases are actively sought, may capture more cases than designs dependent on administrative data alone [21]. Recent studies have made use of administrative healthcare records to identify HD cases [19]. In the absence of case validation, this may lead to the inclusion of miscoded or misdiagnosed HD cases [22].

Factors contributing to heterogeneity in the estimated prevalence can be elucidated by means of a meta-regression. A meta-regression regresses the outcome variable on various predictor variables associated with each study. While its use to explore heterogeneity is common in meta-analysis designs [23], meta-regression models including coefficients, 95% confidence intervals, and a measure of the explanatory power of the model (R^2^ values), have not been formally presented in the HD literature to this date.

In this study, we aim to explore qualitatively and quantitatively, the clinical and methodological heterogeneity between included studies. Heterogeneity is visualized in a forest plot of included studies and summarized by I^2^ values and prediction intervals. Contributing factors to heterogeneity are described and quantitatively evaluated in a meta-regression analysis.

## Methods

The methodology followed the Preferred Reporting Items for Systematic Reviews and Meta-Analysis (PRISMA) protocols checklist. This systematic review is registered on Prospero: CRD42024605294.

### Study selection

We conducted a systematic search of Medline and Embase databases using index terms specific to the prevalence and epidemiology of HD. The search was produced using minor alterations to a search conducted in a previous review (supplementary material) [7]. Two authors (AT/OQ) conducted all stages of the literature review independently. The studies were progressed through the title and abstract screening stage to full record review if they examined either case numbers or prevalence of HD in a defined geographical area. All discrepant selections at title and abstract screening were progressed to the next stage of review for both authors.

At full record review studies were included if: (1) there was sufficient information to calculate a measure of disease burden (point prevalence; period prevalence; cumulative incidence) and binomial confidence interval (at least 2/3 of: the proportional value case numbers/population size, population size, or case numbers), (2) the date of the measure of disease burden was 1993 or later, (3) the full paper was available in English, (4) the population size was at least 100,000, and (5) values reported were based on observational data in a defined geographical region or a representative sample of that region. Since our aim was to describe and quantify the degree of heterogeneity between studies, and not to provide ‘global’ prevalence estimates, we did not require a globally comprehensive dataset to yield valid or meaningful insights. Hence, non-English papers could be reasonably omitted.  Papers were excluded if they were a conference abstract or if they reported a mathematically modeled value. The 1993 date limit was set since this followed the identification of the mutant *HTT* gene, after which time testing for the gene became more routine in clinical practice. The 100,000-population size minimum was set since, given the rarity of HD, reports of disease burden in populations below this size would provide unreliable estimates. This approach is similar to previous reviews [5].

The review papers and included articles had their references searched for further papers. Discrepancies in final study selection were discussed between three authors (AT/OQ/MS) until consensus on inclusion was reached. Referencing software Zotero 6.0.37 was used.

### Data extraction

Manual data extraction for all studies was undertaken independently by two authors (AT/OQ). Data extracted included date of study, number of cases, population size, case ascertainment method, case definition, and location (including continent). Where one of the ‘number of cases’ or the ‘total population size’ was not available, this was extrapolated from back-calculation. Where back-calculation was not possible, but there were reliable external sources of population size estimates (e.g., census records), these external sources were used. For studies presenting multiple point prevalence values at different time points, the case numbers and population size at each time point was extracted. The studies were grouped into three categories for the estimate of HD burden: point prevalence, period prevalence, or cumulative incidence. Case ascertainment methods were broadly categorized into ‘passive’ and ‘passive and active case finding’.

Data extracted was supplemented with online searches for the median age of the population studied and the Healthcare Access Quality Index (HAQI) score of the country. The HAQI score is an index measure which scores countries on a range from 0 to 100 based on both the quality of the healthcare provided and the ease of access for the general population. Where the population of interest was a region within a country, the median age for that country was applied. Any discrepancies in data extraction were discussed between all authors until consensus reached. Study details are available in the supplementary material.

### Risk of bias

We assessed the risk of bias of each study using the Joanna Brigg’s Institute (JBI) critical appraisal tool for prevalence studies [24]. This tool scores studies on their sampling, diagnostic, and statistical methodologies. Scores for each study are available in the supplementary material.

### Data analysis

All studies were included within the comparative qualitative and descriptive analysis. For the quantitative data synthesis (meta-analysis and meta-regression), only a smaller subsection of these studies were included. The studies were included in the quantitative data synthesis if: (1) the estimate of disease burden calculated was a point prevalence, and (2) it was the most recent study within that region. Only including the most recent studies avoided the issue of patients being ‘counted twice’.

Data analysis and synthesis were undertaken using the ‘meta’ package in R version 2023.12.1 + 402. Studies are visually presented in a forest plot grouped by region. Studies were synthesized using a random effect meta-analysis (generalized linear mixed model (GLMM)) and 95% prediction intervals were produced. Prediction intervals describe the range of values within which 95% of similar studies are expected to lie. They are more conservative than confidence intervals since they incorporate study heterogeneity into the range described. Where no heterogeneity is present, prediction intervals coincide with confidence intervals [25]. Prediction intervals for regional subgroups are only presented where *n* ≥ 10 [11].

The meta-regression was conducted using predictor variables agreed between authors a priori*.* These included: continent of study, number of years since 1993, HAQI score, median age of population, and case ascertainment method. As discussed in the introduction, these variables have been shown to be related to overall HD prevalence within a population. Unlike conventional regression analysis, meta-regression incorporates two additional error terms to account for sampling error in studies, and between-study heterogeneity. Meta-regression was conducted using the weighted least squares method.

### Sensitivity analysis

The meta-analysis and meta-regression were repeated with differences in the definition of geographical regions (using the World Bank classification) and excluding studies relying on administrative diagnostic codes for HD diagnosis. The meta-regression was also repeated using fewer predictor variables (year post-1993 and continent).

## Results

### Literature search

**Fig. 1:**
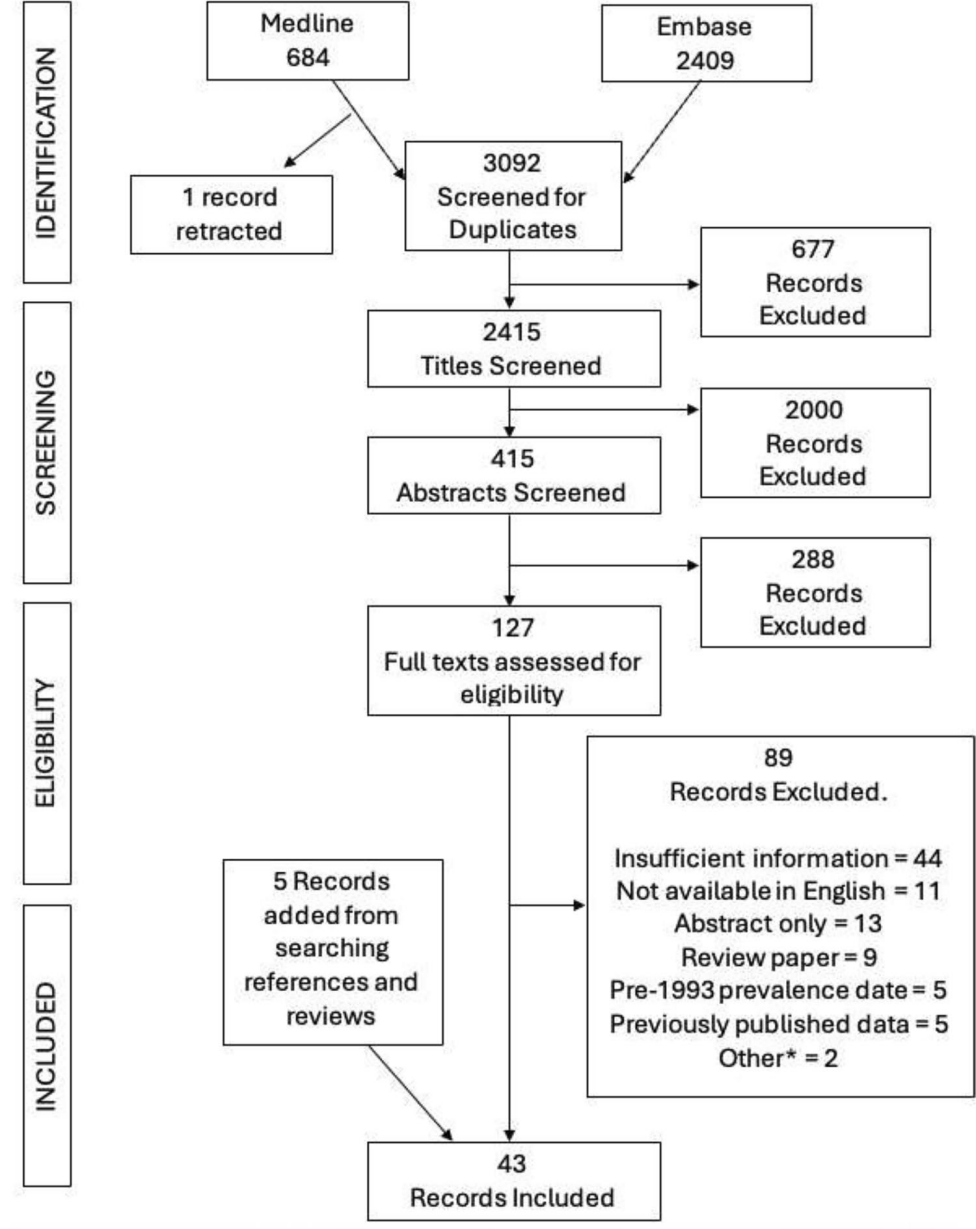
Flowchart of screening and selection process of final papers for inclusion in the systematic review. *Other population size < 100,000 (*n* = 1) and a non-representative sample of a geographical region (*n* = 1)

Medline and Embase databases were searched on 28th October 2024 (Fig. 1). A total of 3093 citations were identified, 2409 in Embase and 684 in Medline. 677 of these records were excluded as duplicates. The titles were examined for relevance to HD prevalence, with 2000 records excluded at this stage. The abstracts of 415 records were reviewed, with 288 excluded. 127 records had their full texts examined, with 89 records excluded. The main reasons for exclusion included: insufficient information to calculate a prevalence value and binomial confidence interval (*n* = 44), paper not available in English (*n* = 11), and the paper being an abstract only (*n* = 13). Other reasons included: paper being a review (*n* = 9), a pre-1993 prevalence date (*n* = 5), estimate of disease burden based on previously published data (*n* = 5), population size < 100,000 (*n* = 1), and calculated prevalence based on a non-representative sample (*n* = 1). Review papers and included records had their references searched for papers not identified through the search strategy, yielding five additional citations. A total of 43 records were included for analysis. A paper using insurance records in the USA was excluded from our analysis since the author’s acknowledged it was not a representative sample of the population [26]. Other studies using insurance records were included where the sample was considered to be representative of the population [27, 28]. A study on a cluster of HD cases in the Minas Gerais state in Brazil was excluded since the population size was < 100,000 [29]. Vishnevetsky et al.’s 2023 study, while focused on juvenile HD in Peru, also reported numbers of adult HD patients and so was included [30]. Details of the included studies are shown in Table 2.

**Table 2:** Huntington’s Disease Prevalence Studies

Region	Prevalence date	Sources of cases	Diagnostic criteria	Population size	Number of cases	Cases per 100,000 population (95% CI)	Estimate type	References
Europe								
Croatia	2002	Unspecified	Symptomatic + genetic confirmation	4,492,049	44	0.98 (0.73, 1.32)	Unclear	Hećimović et al. [31]
Cyprus	2015	MR, ACF	Symptomatic + genetic confirmation	840,517 (calculated)	39	4.64 (3.38, 6.36)	Point prevalence	Demetriou et al. [32]
Denmark	2014	DR, ACF	On disease register + clinically manifest	5,630,000*	329	5.84 (5.24, 6.51)	Point prevalence	Gilling et al. [33]
Finland	2010	MR	Administrative read code	5,377,358	114	2.12 (1.76, 2.55)	Point prevalence	Sipilä et al. [34]
Finland	2020	MR	Administrative read code	5,500,000	142	2.58 (2.19, 3.04)	Point prevalence	Sipilä and Majamaa [35]
Germany	2015–2016	IR	Administrative read code	3,325,638	308	9.26 (8.28, 10.36)	Period prevalence	Ohlmeier et al. [27]
Greece	2008	Lab	Symptomatic + genetic confirmation	10,964,020	594	5.42 (5, 5.87)	Cumulative incidence (1995–2008)	Panas et al. [36]
Iceland	2007	MR, ACF	Symptomatic + family history or genetic confirmation	311,114	3	0.96 (0.18, 2.98)	Point prevalence	Sveinsson et al. [37]
Italy (Ferrara)	2014	MR	Symptomatic + genetic confirmation	354,673	15	4.23 (2.5, 7.04)	Point prevalence	Carrassi et al. [38]
Italy (Molise)	2013	MR, DR, ACF	Clinical phenotype	313,341	34	10.85 (7.72, 15.21)	Point prevalence	Squitieri et al. [39]
Italy (South Sardinia and Cagliari)	2017	MR, Lab, ACF	Symptomatic + genetic confirmation	785,785	47	5.98 (4.48, 7.97)	Point prevalence	Muroni et al. [40]
Malta	1994	MR, ACF	Unspecified	339,173	40	11.79 (8.62, 16.1)	Point prevalence	Gassivaro et al. [41]
Northern Ireland	2001	DR, ACF	Symptomatic + ≥ 36 CAG repeats	1,698,113 (calculated)	180	10.6 (9.16, 12.27)	Point prevalence	Morrison et al. [42]
Russia (Vladimir Oblast)	1994–1999	MR, DR	Clinical + genetic confirmation	1,622,900	31	1.91 (1.34, 2.72)	Period prevalence	Baryshnikova et al. [43]
Scotland (North)	2020	MR	Symptomatic + ≥ 36 CAG repeats	893,440	130	14.55 (12.25, 17.28)	Point prevalence	Kounidas et al. [22]
Slovenia	2006	MR, DR, ACF	Symptomatic + > 36 CAG repeats	2,011,614	104	5.17 (4.26, 6.27)	Point prevalence	Peterlin et al. [44]
Spain (Navarre)	2017	MR, ACF	Clinical + > 35 CAG repeats or positive family history	647,554	32	4.94 (3.48, 7)	Point prevalence	Vicente et al. [45]
Sweden	2018	MR	Administrative read code	10,230,185	1039	10.16 (9.56, 10.79)	Point prevalence	Furby et al. [46]
Sweden (Jamtland)	2015	MR	Administrative read code	126,765	28	22.1 (15.15, 32.06)	Point prevalence	Roos et al. [47]
Sweden (Uppsala)	2015	MR	Administrative read code	348,942	17	4.9 (2.98, 7.87)	Point prevalence	Roos et al. [47]
UK	2018	MR	Administrative read code	2,593,243	239	9.22 (8.12, 10.46)	Point prevalence	Furby et al. [19]
UK	2010	MR	Administrative read code	4,683,669	435	9.29 (8.45, 10.2)	Point prevalence	Douglas et al. [48] and Evans et al. [49]
UK	2011	MR	Administrative read code	2,964,386	177	5.97 (5.15, 6.92)	Point prevalence	Sackley et al. [50]
Wales (South East)	1994	DR, ACF	Clinical + genetic	1,393,900	86	6.17 (4.99, 7.63)	Point prevalence	James et al. [51]
North America								
Canada (Alberta)	2018–2019	MR	Administrative read code	3,183,874	297	9.33 (8.32, 10.45)	Point prevalence	Shaw et al. [9]
Canada (British Columbia)	2012	MR, ACF	Symptomatic + Genetic Confirmation or Strictly Clinical	4,609,659	631	13.69 (12.66, 14.8)	Point prevalence	Fisher et al. [52]
US (Navajo Nation)	2006	MR	Administrative read code	217,158	0	0 (0, 2.14)	Point prevalence	Gordon et al. [53]
South America								
Brazil	2016	Lab, ACF	Symptomatic + molecular diagnosis	11,297,297 (calculated)	209	1.85 (1.62, 2.12)	Point prevalence	Castilhos et al. [54]
Chile	2019	MR, ACF	Symptomatic + > 35 CAG repeats or positive family history	19,107,216	138	0.72 (0.61, 0.85)	Point prevalence	Solís-Ańez et al. [55]
Mexico (Mexico City)	2008	MR	Clinical or molecular diagnosis	7,950,000 (calculated)	318	4 (3.58, 4.47)	Cumulative incidence (1975–2008)	Alonso et al. [56]
Peru	2000–2018	MR	Genetically confirmed	32,203,944*	475	1.47 (1.35, 1.61)	Period prevalence	Vishnevetsky et al. [30]
Venezuela	2006	Lab	Symptomatic + expanded *HTT* allele	27,800,000 (calculated)	88	0.32 (0.26, 0.39)	Cumulative incidence (1988–2006)	Paradisi et al. [57]
Asia								
Israel	2018	IR	Administrative read code	1,580,816	69	4.36 (3.44, 5.53)	Point prevalence	Gavrielov-Yusim et al. [28]
Oman	2019	MR, ACF	Clinical or genetic confirmation	556,731	41	7.36 (5.4, 10.02)	Point prevalence	Squitieri et al. [58]
Japan (San-In area)	1993	MR	Symptomatic + expanded *HTT* allele	1,387,000	9	0.65 (0.32, 1.25)	Point prevalence	Nakashima et al. [59]
South Korea	2013	IR	Administrative read code	51,141,463	197	0.39 (0.33, 0.44)	Point prevalence	Kim et al. [60]
South Korea	2019	IR	Administrative read code	51,849,861	1521	2.93 (2.79, 3.08)	Cumulative incidence (2010–2019)	Lee et al. [61]
Taiwan	2007	IR	Administrative read code	23,000,000	97	0.42 (0.35, 0.51)	Point prevalence	Chen et al. [62]
Oceania								
Australia (New South Wales)	1996	DR, MR, ACF	Symptomatic + positive DNA test or positive family history	6,038,696	380	6.29 (5.69, 6.96)	Point prevalence	McCusker et al. [63]
Australia (Victoria)	1999	DR, Lab	Unspecified	4,736,000	382	8.07 (7.3, 8.92)	Point prevalence	Tassicker et al. [64]
Africa								
Cameroon	2012–2014	MR	Unspecified	3,380,276	2	0.06 (0, 0.23)	Period prevalence	Cubo et al. [65]
Morocco (Rabat-Salé)	2014	MR	Symptomatic + genetic confirmation	2,000,000	21	1.05 (0.68, 1.62)	Unclear	Bouhouche et al. [66]
South Africa	2014	Lab	Referred for testing + HTT expansion	51,207,283 (calculated)	384	0.75 (0.68, 0.83)	Cumulative incidence (1995–2014)	Baine et al. [67]

*DR* disease register, *MR* medical records, *ACF* active case finding, *IR* insurance records. *Taken from World Bank population estimates

### Study heterogeneity

Most included studies were undertaken in Europe (24/43; ~ 56%). Asia and South America had six and five included studies respectively, Africa and North America both had three included studies, while Oceania had only two included studies.

There was variation in how cases were sourced. Most studies used medical records for at least part of their case sourcing (27/43; ~ 63%). A HD register was used in seven included studies. Five studies used diagnostic codes from insurance records, while six studies included records from genetic laboratories. Fourteen studies benefitted from an active case finding component, typically searching for secondary cases within families [58]. In one study, the source of cases was unclear [31].

There was variation in how HD cases were defined. Just over half of included studies incorporated clinical features with genetic confirmation into their case definition (23/43; ~ 53%). Thirteen studies used administrative codes alone to identify HD diagnoses—of these only two detailed attempts to validate the diagnosis. Case validation in these studies involved clinical review of medical records with HD diagnostic read codes to confirm the diagnosis [34, 47]. An insurance-based study in Israel did limit their source of administrative records to either a neurologist diagnosis, or a chronic diagnosis, to improve the specificity of their case identification [28]. In three studies, the case definition was unclear.

Most of the included studies used the whole population size to describe the ‘population at risk’. However, an administrative based study in the UK, an insurance-based study in Israel, and a study in Croatia, included only adults aged ≥ 18 years of age [19, 28, 31], while a Canadian study included only adults ≥ 21 years [9]. The prevalence of juvenile HD is known to be far lower than adult-onset [48].

There was differential inclusion of incident and/or prevalent HD cases in the numerator of the disease burden estimates between studies, as well as whether efforts were made to exclude deceased patients on the prevalence date. A point prevalence value could be calculated in most studies (34/43; ~ 79%). Period prevalence could be calculated in four of the studies. The period prevalence range varied between two and 18 years. Most of the remaining studies (5/43; ~ 12%) appeared to include only incident cases diagnosed over the study period. The range of study periods for which cumulative incidence rates were reported was between 9 and 33 years. In two studies it was unclear how the prevalence value reported had been produced [31, 66].

There was variation in study quality as assessed using the JBI critical appraisal tool [24]. Relatively few studies dropped points for their sample frame (6/43), sampling method (8/43) or sample size (1/43). Larger numbers of studies dropped points on the validity of methods used to diagnose the condition (29/43)—for instance, relying on administrative codes alone—and whether they clearly stated the numerator and denominator in their reporting of results (33/43).

### Meta-analysis

**Fig. 2:**
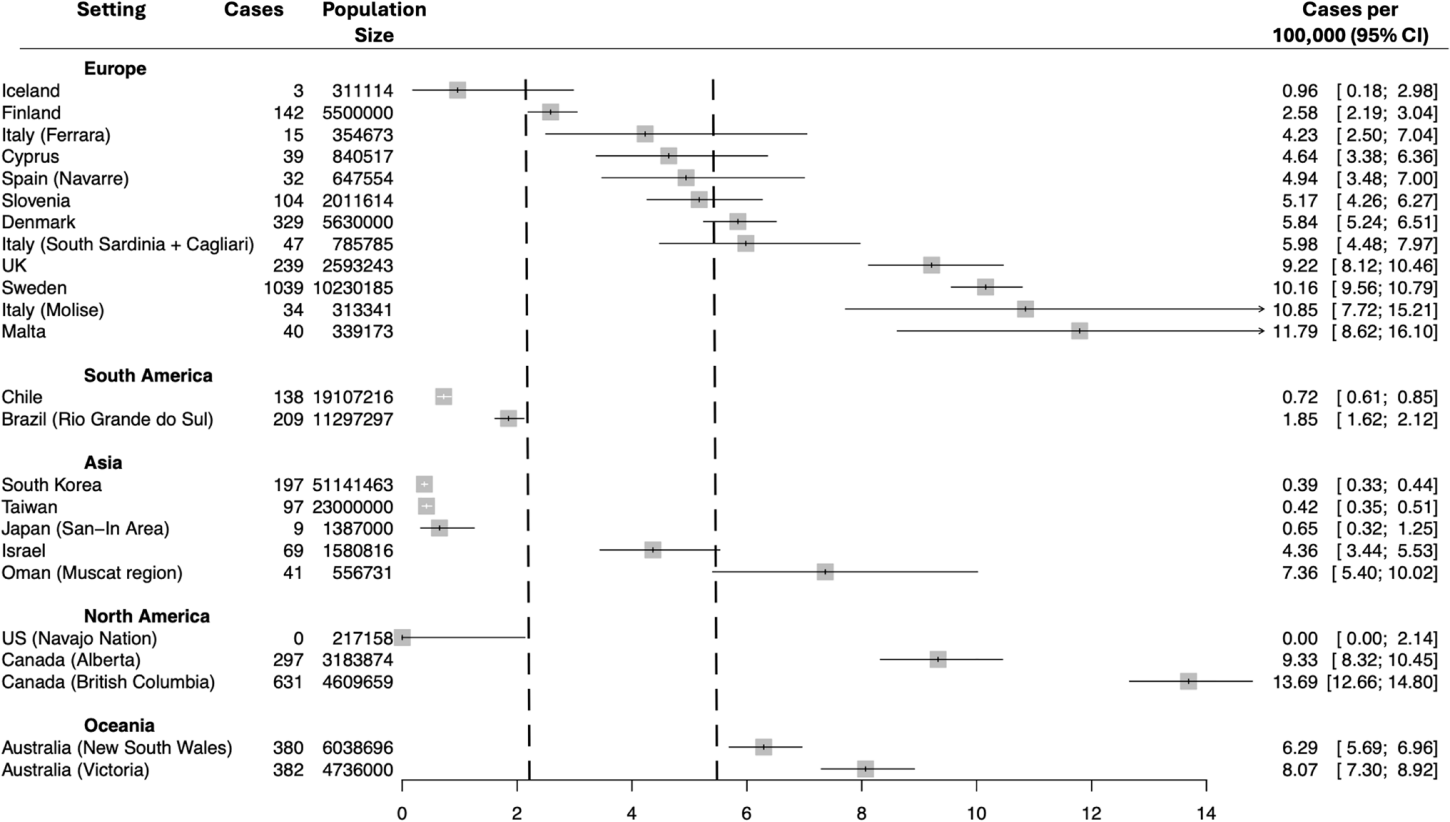
Forest plot presenting HD point prevalence estimates grouped by region. Error bars are 95% confidence intervals. Checked lines are the lower and upper 95% confidence limits for the pooled point prevalence estimate. *CI* confidence interval

Study heterogeneity can be visualized in the forest plot in Fig. 2. Only studies for which a point prevalence value could be calculated were included in the quantitative synthesis and forest plot. Additionally, older studies were excluded where a more recent study in a geographical area was available. In the UK, Furby et al.’s 2022 study was preferred to Kounidas et al.’s 2021 study in Scotland, despite it having an older prevalence date, since it covered a broader and more representative sample of the whole UK population [19, 22]. Studies in other devolved nations in the UK were excluded since the Clinical Practice Research Datalink (CPRD) used in Furby et al.’s study includes practices based in all devolved nations. The final number of populations included in the quantitative synthesis was *n* = 24.

Significant heterogeneity was present both within and between regional subgroups. The overall pooled 95% prediction interval for all studies ranged between 0.32 and 37.55 HD cases per 100,000. Large I^2^ values were reported for all regional subgroups, with a wide 95% prediction interval in Europe (1.64, 19.18). The prediction intervals in other regions are not presented since the subgroup sample size was insufficient. Given the significant heterogeneity present, we do not report an overall pooled point prevalence value, nor pooled point prevalence values for the regional subgroups. The 95% confidence interval of the pooled prevalence value (2.19, 5.46) is presented as the checked lines on the forest plot. Only 10/24 included studies had 95% CIs that overlapped with the 95% CI of the pooled estimate, emphasizing the heterogeneity between study findings.

There is a large range of point prevalence values reported within European populations. Studies in Iceland and Finland reported low point prevalence values (0.96 and 2.58 per 100,000 respectively) [35, 37], while higher point prevalence values were reported in small studies in Italy and Malta [39, 41], and a large administrative-based study in Sweden [46].

The other continents had relatively few studies for inclusion. Asian studies included three in South-East Asia (Japan, South Korea, Taiwan) with similar reported point prevalence values (0.39–0.65 per 100,000) [59, 60, 62], while two Asian studies in the Middle Eastern region had higher point prevalence values reported (4.36–7.36 per 100,000) [28, 58]. In North America a study based on the Navajo Nation found no cases of HD [53], while studies in Canada found high point prevalence values similar to those reported in some of the higher prevalence areas in Europe [9, 52].

### Meta-regression

Table 3 presents the meta-regression analysis. Both Asian and South American based studies had a significantly reduced point prevalence compared to the European reference group. Prevalence in the Asian based studies were estimated at about a quarter of the European reference group, while South American studies were around a twentieth. Studies in Oceania and North America were not statistically significantly different compared to Europe.

**Table 3:** Meta-regression results

Variables	Exponentiated coefficients (CI, 95%)	Standard error	*P* value
Continent			
Europe	1 (ref)	NA	NA
Asia	0.26 (0.09, 0.78)	0.51	0.019
South America	0.05 (0.01, 0.3)	0.86	0.003
North America	1.1 (0.37, 3.25)	0.51	0.855
Oceania	4.45 (0.92, 21.5)	0.74	0.062
Median Age	1 (0.91, 1.11)	0.05	0.987
Year post-1993	1.07 (1.01, 1.13)	0.26	0.022
Case Ascertainment Method			
Passive	1 (ref)	NA	NA
Passive and Active Case Finding	1.97 (0.92, 4.24)	0.36	0.078
Healthcare Access and Quality Index (HAQI) score	0.94 (0.85, 1.04)	0.05	0.228
Constant	0.001	3.4	0.072
R^2^	0.62		

Each year post-1993 was associated with approximately a 7% increase in the point prevalence of HD. The HAQI scores of the study countries was not associated with differences in the point prevalence of HD, and neither was the median age of the underlying population. While our model co-variates did explain a proportion of the heterogeneity between studies (*R*^*2*^ = 0.62), nearly 40% of the heterogeneity between studies remained unexplained.

### Prevalence over time

**Fig. 3:**
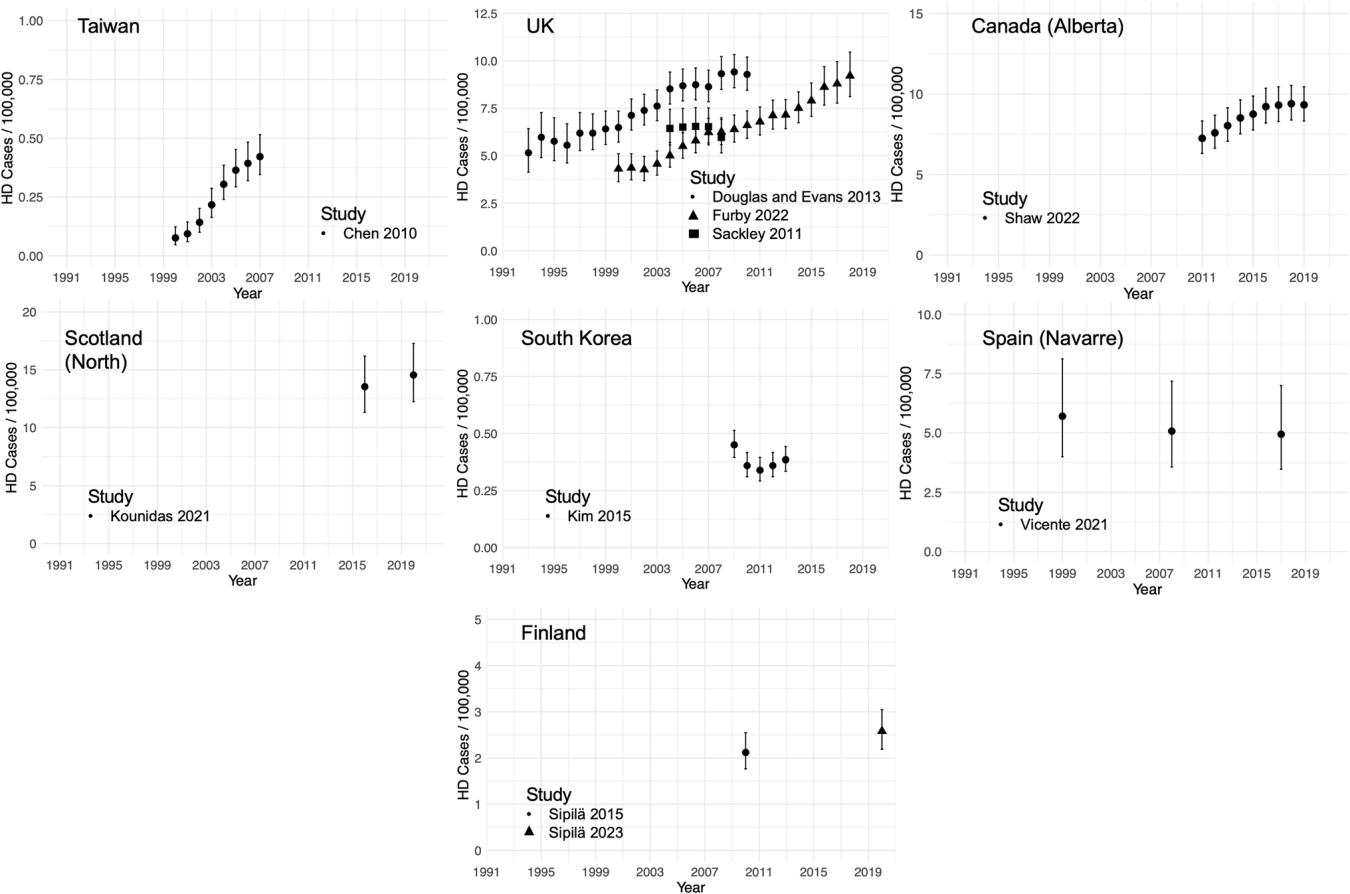
Point prevalence estimates over time in Taiwan, the UK, Canada (Alberta), Northern Scotland, South Korea, Spain (Navarre) and Finland. Error bars are 95% confidence intervals

For some geographical areas there were studies estimating point prevalence values at different points in time. The changes in prevalence over time in these areas are presented in Fig. 3.

The estimated point prevalence appears to have risen over time in: Taiwan, the UK, and the Alberta region of Canada [9, 19, 49, 50, 51, 62]. Four studies (the Douglas et al. and Evans et al. studies were combined to include both juvenile and adult-onset HD) using administrative primary care datasets are included within the UK region, with each study claiming to be based on a representative sample of the UK population. It is notable that in 2004 there is little to no overlap in the UK prevalence estimates between studies, despite apparent similarity of methods and large sample sizes. Further, while Furby et al.’s 2022 study is consistently lower than the other UK HD prevalence estimates presented, since this study actually reports a prevalence in only the ≥ 18-years-old adult population, we would have expected this study to estimate larger prevalence values than the other UK studies [19].

There is little apparent change in estimated prevalence between the time points measured in: Scotland (Northern region), South Korea, Finland, and the Navarre region of Spain [22, 34, 35, 45, 60]. To understand changes in the estimated prevalence of disease over time, it is important to consider changes in both the incidence and mortality of disease over the same period. Of the papers presented in Fig. 3 only Vicente et al.’s 2021 study presented contemporaneous incidence and mortality data [45]. Five of the included studies did present data on incidence alone [9, 19, 50, 60, 62]. Increasing point prevalence mirrors increasing incidence in Taiwan [62]. However, in the UK and Canada apparent rises in the estimated point prevalence values occurred against a background of fluctuating incidence levels, possibly indicating that improvements in patient longevity have driven the changes [9, 19].

### Sensitivity analysis

The meta-analysis and meta-regression were repeated with an alternative regional grouping (World Bank classification). We also repeated the meta-analysis and meta-regression excluding studies based on administrative data alone and repeated the meta-regression including fewer predictor variables (supplementary material).

Significant heterogeneity remained, with large regional I^2^ values and wide overall and European (or ‘Europe and Central Asia’) 95% prediction intervals. Adjusting the region definitions to the World Bank classification meant that none of the adjusted meta-regression coefficients were statistically significant at the 5% level. When administrative code-based studies were excluded the adjusted meta-regression coefficients were grossly similar, with South American and Asian studies being associated with lower prevalence of HD, while each additional year post-1993 was associated with a 7% increase in HD prevalence. Results in our meta-regression model with fewer predictor variables (continent and year post-1993) were largely similar to our presented model, although the model’s explanatory power was lower (*R*^*2*^ = 0.5).

## Discussion

### Heterogeneity between regions

The large heterogeneity between studies in different regions is demonstrated by the wide overall pooled prediction interval incorporating all studies. The overall 95% prediction interval ranged between 0.32 and 37.55 HD cases per 100,000—an over 100-fold difference in possible point prevalence values. While prediction intervals had been suggested as a mechanism for more conservatively synthesizing studies where true heterogeneity is expected [14], the overall prediction interval produced in the context of such heterogeneity is likely too wide to be meaningful. The adjusted meta-regression analysis demonstrated reduced HD point prevalence within both the South American and Asian continents, compared with the European reference group (around a quarter in Asian based studies; a twentieth in South American based studies). Lesser HD prevalence in Asian populations is widely recognized and likely reflects a combination of genetic factors: protective genetic haplotypes, reduced mean CAG repeat length in the general population, and CCG polymorphisms [68]. The small sample of South American studies (*n* = 2) limits conclusions based on this result.

### Heterogeneity within regions

Heterogeneity between studies in the same region is demonstrated by large regional I^2^ values (all > 90%). The prediction interval for the European subgroup ranged between 1.64 and 19.48 HD cases per 100,000. This significant within region study heterogeneity indicates that ‘lines on a map’ may be a poor proxy for genetic similarity between populations within defined geographic regions. Indeed, Gordon et al.’s 2016 paper in the Navajo Nation in the US found no cases of HD, while papers based in Canada reported point prevalence values closer to the high prevalence populations in Europe. While these populations co-exist in the geographically defined North American continent, genetic differences between the different races studied will contribute to the differences in point prevalence estimates reported. Conversely, while no cases were reported in the Navajo nation it is recognized that founder effects and clustering of large families in certain communities may lead to an observed HD prevalence that is significantly greater than surrounding areas [29]. Further, where HD burden is broken down by race, as in Baine et al.’s 2016 paper reporting HD cases in South Africa, racial differences are a much more significant predictor of genetic similarity, and consequently HD burden, than geographical area [67]. An approach which pooled racially disparate populations into overall regional populations would lose important nuance in differences in HD prevalence between different racial groups. This is perhaps clearest in the ‘Asian’ studies within this systematic review. While studies based in South-East Asia reported largely similar point prevalence values (Taiwan, South Korea, and Japan) [59, 60, 62], when combined with the Israel and Oman studies in the Middle East under the ‘Asia’ umbrella, the heterogeneity dramatically increases [28, 58].

### Heterogeneity over time

There is heterogeneity in point prevalence values observed at different times within our inclusion window. The adjusted meta-regression analysis demonstrated a positive association between the number of years since 1993 (the start of our inclusion window) and the prevalence date. On average, for each year after 1993, there was a 7% increase in the estimated HD point prevalence. Increasing estimates over time is also demonstrated in the included studies reporting point prevalence values at different time points (Fig. 3). In Taiwan, the UK, and the Alberta region of Canada, there are clear increases in the estimated point prevalence over time. Increasing estimates of point prevalence over time was not universal: studies in Spain and South Korea, did not demonstrate increases [45, 60]. Suggested factors driving increases in the estimated point prevalence include an increase in expected survival of HD patients from diagnosis, and reduced stigma around disease diagnosis [20]. Additionally, where studies are repeated in the same region at different time points an increase in point prevalence may be noted due to improved case ascertainment over time (for instance, diagnosing secondary cases in families with established heterozygosity for the expanded *HTT* allele). Moreover, widening access to genetic testing may have facilitated further diagnoses.

While increases in disease prevalence can follow increases in disease incidence, Furby et al.’s 2022 paper in the UK reported unchanged HD incidence rates over the 19-year study period [19]. It is also recognized that some under ascertainment on the prevalence date may occur due to the presence of symptomatic individuals who have not yet been diagnosed. Attempts to address this cause of under ascertainment have been made by using a prevalence date just before, or early in, the survey period [42, 63]. Patients symptomatic on the prevalence date but not diagnosed until later in the study period would, therefore, be included. We recommend that studies reporting a single estimated point prevalence value use remote prevalence dates where possible or if using estimates over time, include contemporaneous data on incidence and mortality.

### Heterogeneity in estimates of disease burden

There are significant differences in the estimates of HD burden that can be calculated from studies reporting HD cases. Of the 43 studies included within this systematic review, four could be used to produce estimates of period prevalence, while estimates of the cumulative incidence of HD cases could be calculated in five studies. Point prevalence values could be calculated in 34 studies. For valid quantitative synthesis, these distinctions in estimate types matter. Period prevalence values include all cases (prevalent and incident) during a time period, without excluding any cases that died during the study period. Consequently, the estimated number of cases is inflated (since deaths are not removed) and the prevalence value is also inflated. This is exemplified in Shaw’s 2022 Canadian study reporting yearly point prevalence values for the period 2014–2019, and a period prevalence value across the same six years [9]. While the yearly point prevalence values range around 8–9/100,000, the period prevalence value reported over the same period is about a third higher at 12.15/100,000. Conversely, cumulative incidence values may provide either inflated or deflated approximations of point prevalence. Although case numbers are reduced since prevalent cases are not included, failing to exclude deceased patients will inflate case numbers reported. Consequently, whether or not a cumulative incidence estimate is greater or lesser than a point prevalence estimate depends on the length of the study period. Previous meta-analyses of HD prevalence have quantitatively synthesized studies reporting estimates of HD burden across all three categories described, without distinction or comment [7, 8]. We recommend that studies intending to report a measure of burden of HD make clear the estimate type reported.

### Heterogeneity in case ascertainment

There are significant differences between studies in how HD cases were ascertained. Thirteen of the included studies used administrative read codes alone to find HD cases. There is concern in the literature around the validity of using administrative codes for this purpose, which is dependent on the quality of the clinical coding [45]. Indeed, inflation of case numbers due to false positives in administrative codes may be significant. Sipilä et al.’s 2015 study in Finland incorporated clinical review of medical records into their case ascertainment process [34]. Of the 399 records identified as having an HD diagnostic code, only 207 cases were validated as HD following chart review. Further, four UK based studies using administrative records from primary care databases all reported differing point prevalence estimates for the same time periods [19, 48–50]. While the rarity of HD makes traditional door-to-door survey methods for case finding impractical in most settings, it is important that the use of routine administrative data in case finding is rigorous and validated. Further attempts to assess and improve the validity of administrative data in epidemiological studies of HD prevalence should be a priority for future research. Incorporating multiple methods of ascertainment into the study design provides a mechanism for both validating cases (through triangulation of evidence) and for identifying cases that would otherwise have been missed [45, 52]. Under ascertainment of HD will apply to varying degrees among all included papers, particularly since a significant proportion of HD patients may present with psychiatric symptoms in advance of the more typical motor phenotype [69].

### Heterogeneity in defining the population at risk

Differences in how the population at risk of HD is defined, may also lead to inflation of the estimated HD point prevalence in some contexts. Within this systematic review three papers included only cases of adult-onset HD, and therefore, included only the adult population within the population at risk [9, 19, 28]. Since juvenile onset HD makes up only a small fraction of overall HD cases, ‘adult-only’ point prevalence values would be expected to be higher than the estimated prevalence in the whole population [70].

### Implications for further research and practice

While the validity of pooled prevalence values, and the utility of prediction intervals—in the context of high heterogeneity—have been questioned in this paper, there is significant value in continuing to undertake descriptive systematic reviews of HD prevalence, particularly as new studies and new methodologies for case ascertainment emerge. We recommend that authors publishing studies of HD prevalence consider and describe how the sources of heterogeneity we describe here may apply to their study. Indeed, developing consensus on the minimum standards and reporting requirements for HD prevalence studies could offer significant benefit to the academic community, and reduce the diversity in methodologies that currently limits meaningful quantitative synthesis.

Further, for policy makers working in a context where HD prevalence is both unknown and an epidemiological study is not feasible, we advise a ‘nearest’ population approach, utilizing findings from systematic reviews of HD prevalence. This approach would take the prevalence within ethnically similar populations as a useful guide for the possible prevalence range within their own population. For instance, public health professionals in China would find more benefit in considering HD prevalence in nations such as Taiwan, rather than a nominally ‘global’ pooled prevalence value. Such considerations could be further developed by considering additional factors such as the presence of significant migrant populations within the region.

### Strengths and limitations

This systematic review benefits from the large number of studies that have been undertaken since the identification of the mutant *HTT* allele in 1993. This enables meaningful evaluation of the different sources of heterogeneity between studies and settings. In comparison to previous reviews which speculate qualitatively on the contribution of different factors to heterogeneity [4], our meta-regression approach enables quantification of the relative contribution of different factors to HD prevalence.

However, caution must be advised in interpreting our meta-regression coefficients. The reduced sample size for our quantitative analysis (*n* = 24) means our regression model may be underpowered to find a true association where one exists, while model overfitting may also occur. Nevertheless, statistically significant predictors of the point prevalence of HD in our adjusted meta-regression model included a lesser point prevalence of HD in South American and Asian populations compared with the European reference population, and an increase in the point prevalence of HD in the years after 1993. These findings are epidemiologically plausible and are consistent with findings in other studies [6–8, 68]. A larger sample size of included studies may have been available had we been able to include non-English studies within our review. The exclusion of non-English papers may mean there are elements of heterogeneity between published studies that have not been captured within our review, and that our estimates of the scale of heterogeneity between published estimates, although already large, may be underestimates of the true heterogeneity in the published literature globally.

## Conclusion

This systematic review describes the significant heterogeneity between studies used to estimate the burden of HD in different settings. Sources of clinical heterogeneity include differences between regions and between different population groups within regions, and differences in HD prevalence over time. Sources of methodological heterogeneity include differences in case definitions and case ascertainment methodologies, as well as differences in the estimates of disease burden produced. Such heterogeneity currently limits the validity and meaningfulness of a meta-analysis approach, while pooled prediction intervals are likely too wide to be meaningful for researchers and implementers of health policy. Developing an accepted consensus on the minimum standards and reporting requirements for HD prevalence studies could help reduce the methodological heterogeneity between future studies, enabling more valid and meaningful quantitative synthesis in future.

## Data Availability

The data supporting the findings of this study can be found in the supplementary material of this article.
